# An explanatory model of depressive symptoms from anxiety, post-traumatic stress, somatic symptoms, and symptom perception: the potential role of inflammatory markers in hospitalized COVID-19 patients

**DOI:** 10.1186/s12888-022-04277-4

**Published:** 2022-10-10

**Authors:** David Villarreal-Zegarra, Rubí Paredes-Angeles, Nikol Mayo-Puchoc, Ana L. Vilela-Estada, Anthony Copez-Lonzoy, Jeff Huarcaya-Victoria

**Affiliations:** 1 Escuela de Medicina, Universidad César Vallejo, Trujillo, Peru; 2 Instituto Peruano de Orientación Psicológica, Lima, Peru; 3 Unidad de Investigación en Bibliometría, Universidad San Ignacio de Loyola, Lima, Peru; 4 PSYCOPERU Peruvian Research Institute of Educational and Social Psychology, Lima, Peru; 5 Escuela Profesional de Medicina Humana, Universidad Privada San Juan Bautista, Filial Ica, Peru; 6 Departamento de Psiquiatría, Servicio de Psiquiatría de Adultos, Unidad de Psiquiatría de Enlace, Hospital Nacional Guillermo Almenara Irigoyen, Lima, Perú

**Keywords:** COVID-19, Inflammation, Depression, Anxiety, Post-traumatic stress, Peru

## Abstract

**Background:**

The context of the COVID-19 pandemic has harmed the mental health of the population, increasing the incidence of mental health problems such as depression, especially in those who have had COVID-19. Our study puts forward an explanatory model of depressive symptoms based on subjective psychological factors in those hospitalized for COVID-19 with and without biological markers (i.e., inflammatory markers). Therefore, we aim to evaluate the hypotheses proposed in the model to predict the presence of depressive symptoms.

**Method:**

We conducted a cross-sectional study, using a simple random sampling. Data from 277 hospitalized patients with COVID-19 in Lima-Peru, were collected to assess mental health variables (i.e., depressive, anxiety, post-traumatic stress, and somatic symptoms), self-perception of COVID-19 related symptoms, and neutrophil/lymphocyte ratio (NLR) such as inflammatory marker. We performed a structural equation modeling analysis to evaluate a predictive model of depressive symptoms.

**Results:**

The results showed a prevalence of depressive symptoms (11.2%), anxiety symptoms (7.9%), somatic symptoms (2.2%), and symptoms of post-traumatic stress (6.1%) in the overall sample. No association was found between the prevalence of these mental health problems among individuals with and without severe inflammatory response. The mental health indicators with the highest prevalence were sleep problems (48%), low energy (47.7%), nervousness (48.77%), worry (47.7%), irritability (43.7%) and back pain (52%) in the overall sample. The model proposed to explain depressive symptoms was able to explain more than 83.7% of the variance and presented good goodness-of-fit indices. Also, a different performance between the proposed model was found between those with and without severe inflammatory response. This difference was mainly found in the relationship between anxiety and post-traumatic stress symptoms, and between the perception of COVID-19 related symptoms and somatic symptoms.

**Conclusions:**

Results demonstrated that our model of mental health variables may explain depressive symptoms in hospitalized patients of COVID-19 from a third-level hospital in Peru. In the model, perception of symptoms influences somatic symptoms, which impact both anxiety symptoms and symptoms of post-traumatic stress. Thus, anxiety symptoms could directly influence depressive symptoms or through symptoms of post-traumatic stress. Our findings could be useful to decision-makers for the prevention of depression, used to inform the creation of screening tools (i.e., perception of symptoms, somatic and anxiety symptoms) to identify vulnerable patients to depression.

**Supplementary Information:**

The online version contains supplementary material available at 10.1186/s12888-022-04277-4.

## Background

Several studies have reported that COVID-19 patients had experienced various mental health problems (i.e., depression, anxiety, and post-traumatic symptoms) [1–3]. Systematic reviews have identified a high prevalence of depressive symptoms (52%), anxiety symptoms (47%) [4], and symptoms of post-traumatic stress (26.9%) [5] as a result of COVID-19. The evidence suggests that individuals who contracted COVID-19 suffered a negative impact on their mental health, however, this impact was greater in individuals who were hospitalized for COVID-19 [6]. In this way, patients who were hospitalized had a greater negative impact on their mental health due to various clinical factors such as demographics (i.e. sex, age, and proceeding outside of the capital), clinical (i.e. self-perception of the severity of COVID-19, the persistence of COVID-19 symptoms, a history of psychiatric treatment, and history of a family member infected by COVID-19), immune factors (i.e. neutrophil–lymphocyte index greater of 6.5), and psychosocial characteristics (i.e. isolation or quarantine, fear of COVID-19, being discriminate because of COVID) [7–10].

There are two mechanisms of action that may explain the presence of mental health problems in hospitalized patients with COVID-19: biological and psychological responses. Although the neuropsychiatric complications of COVID-19 are under study, there is evidence that inflammatory markers may cause mental health problems, such as depression. An invasion of SARS-CoV-2 to the respiratory tract could induce an acute respiratory syndrome with consequent release of proinflammatory cytokines such as IL-1β and IL-6. Consequently, a systemic immune response in the form of a “cytokine storm” is produced [11]. Moreover, studies have reported these cytokines have increased in various psychiatric disorders (i.e., schizophrenia, depression, and post-traumatic stress) [12]. The relationship between elevated cytokine levels in COVID-19 and mental health problems could indicate that immune/inflammatory pathways are one of the possible mechanisms involved in mental health problems in this infection [12].

The neutrophil–lymphocyte ratio (NLR) is an inexpensive marker calculated through a complete blood count. Its pathogenic role has been studied in a wide variety of diseases [13–16]. Thus, elevated NLR has been related to an increase of cytokines and C-reactive protein (CRP). Elevated NLR levels are also associated with a state of chronic inflammation. Recent meta-analyses have documented the relevance of NLR in psychiatric diseases such as schizophrenia [14], and mood disorders [17]. If the measurement of cytokine performance is not possible, we can indirectly assess an increase in cytokines through an elevation of NLR.

From a psychological point of view, patients hospitalized with COVID-19 experience physical discomfort related to the COVID-19 symptoms themselves and other somatic symptoms, which can lead to stress-induced mental health problems [18, 19]. As a novel and life-threatening disease, COVID-19 can cause fear and stress in patients, especially for those being treated in the isolation ward. Also, the uncertainty regarding the consequences of the infection during the hospitalization may intensify patients’ experiences of panic [20]. Systematic reviews with meta-analysis evidence that the isolation, physical discomfort, and adverse effects of treatment may increase sensitivity among patients around symptoms of the infection, which could lead to worsening of mental health [18, 21–23]. Additionally, stress and anxiety could cause depressive symptoms [24].

**Fig. 1:**
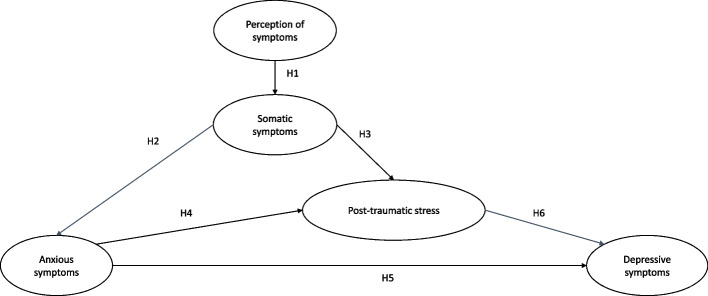
Model tested using structural equation modeling (SEM)

There are studies that separately evaluate the relationship between mental health in hospitalized COVID-19 patients with psychological factors and biological markers. However, few studies investigate the impact of both factors on mental health problems (i.e., depression) in COVID-19 patients. Therefore, we conducted a study to explain the presence of one of the most prevalent mental health problems (i.e., depressive symptoms) from subjective psychological factors (i.e., somatic symptoms, anxiety symptoms, and symptoms of post-traumatic stress) in individuals hospitalized for COVID-19 with and without biological markers (i.e., inflammatory markers) (see Fig. 1).

### Perception of the severity of COVID-19 symptoms influences somatic symptoms (hypothesis 1) (see Fig. 1

Patients' self-perception of the severity of illness (i.e., reducing or increasing symptoms) is related to the severity of mental health problems. A possible explanation is that concerns about their illness or condition add to their psychological burden [25]. Furthermore, the perception of symptoms related to COVID-19 (i.e., fever, cough, trouble breathing) are closely connected to somatic symptoms (i.e., headache, feeling tired, etc.). Therefore, it is also related to other mental health problems.

### Somatic symptoms influence anxiety symptoms (hypothesis 2) and symptoms of post-traumatic stress (hypothesis 3) in patients hospitalized for COVID-19 (see Fig. 1)

It has been evidenced that the prevalence of somatic symptoms is significantly related to psychological outcomes (i.e., anxiety and post-traumatic stress). Evidence shows a high prevalence of moderate or severe anxiety during the COVID-19 pandemic among the general public. In many cases, a common anxiety-induced comorbidity was somatization [26]. In contrast, PTSD is a severe psychological consequence when a person experiences a stressful event as highly traumatic [27]. Indeed, longitudinal studies about PTSD in trauma survivors reported that symptoms of post-traumatic stress establish a more consistent relationship with somatic symptoms over time [28, 29].

### Anxiety symptoms influence post-traumatic stress symptoms (hypothesis 4) in patients hospitalized for COVID-19 (see Fig. 1)

There is much evidence to support the triad of fear, anxiety, and stress. This triad is a fear-induced sequence of responses (being hospitalized for COVID-19) that leads to an anxiety response. This in turn leads to symptoms of post-traumatic stress. Firstly, fear may increase sympathetic nervous system arousal and induce defensive or escape behavior in the face for specific and real threatening stimulus. Anxiety is like fear as an emotional reaction, but unlike fear, the source of threat is unclear. Thus, it is associated with preventive behaviors such as avoidance [30]. Moreover, fear and anxiety are related to the amygdala, which recruits and expresses the memory of these emotions in both animals and humans [31].

The fear caused by COVID-19 can be implicated in mental health problems (i.e., insomnia, increased alcohol and tobacco use, anxiety, among others), such as high infection and death rates, strict public health measures, etc. [7]. In many cases, the excessive exposure to anxiety behaviors triggers post-traumatic stress disorder (PTSD) [27].

### Symptoms of post-traumatic stress and anxiety symptoms influence the presence of depressive symptoms (hypothesis 5 and 6) in patients hospitalized for COVID-19 (see Fig. 1)

The evidence on the relationship between anxiety, depression, and post-traumatic stress is abundant. Studies have identified that anxiety and fear of being hospitalized for COVID-19 can generate a state of acute stress in individuals [32, 33]. Acute stress and symptoms of post-traumatic stress often trigger different mental health problems such as depression in hospitalized patients [20, 34]. Therefore, it is hypothesized that PTSD symptoms precede depressive symptoms (hypothesis 6: symptoms of post-traumatic stress influences depressive symptoms). On the other hand, there is ample evidence that both anxiety and depression are closely related [35, 36] especially in COVID-19 pandemic [37]. Previous studies confirmed this hypothesis, where anxiety, post-traumatic stress, and depression were closely related to each other. Also, anxiety had the greatest influence on the prevalence of depressive symptoms [24, 38] (hypothesis 5: anxiety symptoms influence depressive symptoms).

While studies about mental health in COVID-19 are in ascendant progress, there is a lack of clarity about the functioning of these variables and their subsequent impact on mental health. Therefore, the present study proposes to evaluate these hypotheses to predict the presence of depressive symptoms from subjective psychological factors (i.e., somatic symptoms, anxiety symptoms, symptoms of post-traumatic stress) in those hospitalized for COVID-19 with and without biological markers (i.e., inflammatory markers).

## Methods

### Study design

The study design was a cross-sectional investigation.

### Participants

We used secondary data from Mental Health in COVID-2019 Survivors from a General Hospital in Peru: Sociodemographic, Clinical, and Inflammatory Variable Associations [39]. Participants were individuals with COVID-19 who were discharged from the “Hospital Nacional Guillermo Almenara Irigoyen” between March and September 2020 in Lima, Peru. Inclusion criteria included the following: 18 years or older, having been assessed at their admission and release from the hospital. Participants were excluded as follows: individuals who had missing data in the variables of interest (i.e., anxiety symptoms, depressive symptoms, somatic symptoms, post-traumatic stress symptoms, and NLR) and demographic variables (i.e., age, sex, civil status. degree of education, employment status, partners, relatives with COVID-19).

We calculated sample size with the Epidat v44.2 program (Dirección Xeral de Saúde Pública da Consellería de Sanidade, Galicia, España) and the sample was selected by a simple random sampling from a total number of 1190 participants.

### Setting

The data of this second study were collected for the HNGAI from September to November of 2020. “Hospital Nacional Guillermo Almenara Irigoyen” is classified as a third-level specialized health institute in 2015, and it is the second-largest hospital in the “Seguridad Social de Salud del Perú'' (ESSALUD).

During the COVID-19 pandemic, the health system was focused on the care of COVID-19 patients. Thus, third-level hospitals were responsible for providing hospital beds from their different specialties to these patients due to the high demand for care. COVID-19 was diagnosed by serological and molecular tests.

### Variables and measurement instruments

#### Depressive symptoms

The Patient Health Questionnaire-9 (PHQ-9) is a self-reporting instrument developed to identify possible causes or measure the severity of depressive symptoms within the last two weeks [40]. It is based on the 9 criteria diagnostics from the Diagnostic Statistical Manual of Mental Disorders, five editions [DSM-5]. The items are scored on a four-point scale, ranging from 0 (“not at all”) to 3 (“nearly every day”). Total scores range from 0 to 27 with severity levels of minimal (score 0 to 4), mild (score 5 to 9), moderate (score 10 to 14), moderate-severe (score15 to 19), and severe (score 20 to 27) depressive symptoms. Also, the screening cut-off point of 10 or more is considered as the presence of clinically relevant depressive symptoms [41, 42]. The Spanish version of the PHQ-9 conducted in Peru, developed by Villarreal-Zegarra [43], showed good psychometric properties. In this study, the scale displayed good levels of reliability (Cronbach’s α = 0.88) and validity (See details in Supplementary material 1).

#### Anxiety symptoms

The General Anxiety Disorder- 7 scale (GAD-7), is a self-report scale that assesses the presence or severity of generalized anxiety disorder (GAD) during the 2 weeks before self-application. The items reflect the most prominent diagnostic features of the DSM-5 symptoms criteria for GAD. Response options are scored on a four-point scale: 0 (“not at all”), 1 (“several days”), 2 (“more than half the days”), and 3 (“nearly every day”). Total scores range from 0 to 21 and are categorized as follows: minimal (score 0 to 4), mild (score 5 to 9), moderate (score 10 to 14) and severe levels of anxiety symptoms (score 15 to 21) [44]. In addition, the GAD-7 has a cut-off range of 10 points or more to identify the presence of clinically relevant anxiety symptoms [45–47]. The scale has been translated into Spanish and validated by García-Campayo et al. [48]. In the present study, GAD-7 scale had adequate levels of internal consistency (Cronbach’s α = 0.87) and validity (See details in Supplementary material 1).

#### Somatic symptoms

The Patient Health Questionnaire-15 (PHQ-15) is a scale derived from the full PHQ. It measures 15 somatic symptoms that entail more than 90% of the physical complaints during the past 4 weeks. Items are based on the most prevalent DSM-IV somatization disorder somatic symptoms. The items has three-type Likert response options: 0 (“Not bothered at all”), 1 (“Bothered a little”), and 2 (“Bothered a lot”). Total scores range from 0 to 30 with severity levels of minimal (score 1 to 4), low (score 5 to 9), medium (score 10 to 14), and high (score 15 to 30) somatic symptoms [49, 50]. It presents a cut-off point of 15 to consider clinically significant somatization. The PHQ-15 scale has been translated and validated into Spanish by Ros [51].

Due to differences in some samples in terms of the specific factors: one, two, three [52–54] and four factors [50, 55, 56], we conducted a sub-analysis to assess the psychometric properties of the PHQ-15 through factor analysis and reliability (See the Supplementary material 1). As a result, instead of using the PHQ-15 scale, we decided to use a version of 12 items (PHQ-12), which showed good psychometric properties (Cronbach’s α = 0.83).

#### Symptoms of post-traumatic stress

The Impact of Events Scale-Revised (IES-R) is a self-report scale that measures the degree of suffering caused by a life event, described as a form of subjective stress during the past 7 days. The IES-R has 22 items scored with five-point scale, ranging from: 0 (“not at all”) to 4 (“extremely”). It is categorized in three dimensions: a) Intrusion dimension (e.g. intrusive distressing thoughts, nightmares, feelings, and images), which items are 1, 2, 3, 6, 9, 14, 16, and 20, b) avoidance dimension (e.g. avoidance of feelings, situations or ideas), which items are 5, 7, 8, 11, 12, 13, 17, and 22, and c) hyperarousal dimension (e.g. anger, hypervigilance, irritability, difficulty concentrating), which items are 4, 10, 15, 18, 19, and 21 [57]. The total scores reflect severity levels of distress symptoms as follows: normal (score 0 to 8), mild (score 9- 25), moderate (score 26 to 43), and severe (score 44 to 88) [58]. Moreover, the scale presents a cut-off point of 33 or more that entails clinically relevant symptoms of post-traumatic stress [59]. The scale has been translated and validated in an into Spanish [60]. In the current study, IES-R total score had adequate levels of internal consistency (Cronbach’s α = 0.95) and validity (See Supplementary material 1).

#### Perception of symptoms

Self-perception of COVID-19 related symptoms was assessed through two questions in Spanish. The first question asked about how many symptoms the person self-reported at the time of admission to hospitalization and the second question asked about the number of symptoms the person reported at the time of assessment with the psychological instruments. To determine the self-perception of symptoms, the difference between these two questions was assessed. It was expected that if the number of symptoms increased the person self-perceived that his or her illness worsened (positive values) and if the number of symptoms decreased the person perceived that his or her health status improved (negative values). The symptoms were fever, fatigue, myalgia, cough, dyspnea, odynophagia, rhinorrhea, diarrhea, nausea or vomiting, anosmia, ageusia, headache, dizziness, ataxia, and convulsions.

#### Neutrophil–lymphocyte ratio (NLR)

The neutrophil–lymphocyte ratio (NLR) was obtained from the patients' complete blood counts on admission. It consists of the ratio between the neutrophil count and the lymphocyte count. The NLR was categorized into < 6.5 and ≥ 6.5. This cutoff point was chosen considering its ability to predict mortality in patients with COVID-19 [61].

#### Sociodemographic variables

Information was provided on age, sex, civil status, and employment status. The following questions were also queried: 1) Do you belong to any religion? 2) Have you had a family member with COVID-19? 3) Has any member of your family passed away due to COVID-19? 4) Do you have a previous psychiatric diagnosis? and 5) Have you had a previous psychological treatment?

### Data analysis

#### Descriptive and prevalence

A descriptive analysis of participants was conducted. The prevalence of depressive symptoms (PHQ-9 > 10 or more) [41, 42], anxiety symptoms (GAD-7 > 10 or more) [45, 46], symptoms of post-traumatic stress (IES-R > 33 or more) [59]). We performed a differentiated analysis of the symptoms and indicators of the PHQ-9, GAD-7, IES-R and PHQ-12. The results were stratified based on those with high neutrophil counts (NLR ≥ 6.5), indicating risk of mortality in patients with COVID-19 [61].

#### Relation between variables

Spearman’s Correlation was used to measure the degree of association between variables. We categorized the size of the correlation coefficient as follows: a large (*r* > 0.70), moderate (*r* > 0.50), or small (*r* > 0.30) ratio [62].

#### Structural regression model

Due the data were non-normal (i.e., categorical indicators), we used a structural regression model with the weighted least squares means and variance adjusted (WLSML) estimator [62]. A polychronic correlation matrix for the nature of the items was also used. Four goodness-of-fit indices were evaluated the proposed model for hospitalized participants with high and low NLR: Comparative Fit Index (CFI), Tucker-Lewis's index (TLI), standardized root mean square residual (SRMR), and root mean square error of approximation (RMSEA). Also, its points cohort is as follows: a) CFI and TLI > 0.95 or more, b) SRMR and RMSEA < 0.08 or flew [63, 64]. In addition, we evaluate the R2 of the outcome variable (depressive symptoms) to determine how much variance explains the proposed model [65].

To ensure sufficient statistical power to perform structural regression analysis, it was considered necessary to have at least 200 participants in total [65]. In addition, to maintain the internal validity of the results, we sought to ensure that exposed and unexposed cases with the inflammatory response (i.e., NRL) had similar sizes.

#### Statistical software

All analyses were done in R studio version 4.1.1, with the packages “lavaan”, “semTools” and “semPlot” [66].

## Results

### General characteristics and prevalence

From 319 patients with a diagnosis of COVID-19, we excluded 42 of them as they did not record NLR measurements. Thus, we analyzed data from 277 participants (86.8% of the total number of patients). The average age was 54.2 (± 14.9) years, and most patients were men (61.4%). Two-hundred and twenty-five (81.2%) had at least one family member with COVID-19, and eighty-five (30.7%) had at least one relative die by COVID-19. Most participants did not have a psychiatric diagnosis (93.1%) and did not receive psychological treatment (91%) prior to their COVID-19 infection. Regarding the prevalence of mental health problems, 11.2% was the overall prevalence of depressive symptoms, 7.9% for anxiety symptoms, 2.2% for somatic symptoms, and 6.1% for post-traumatic stress symptoms.

The 48.7% of the participants had a severe inflammatory response, the analysis differentiated by those with and without severe response can be seen in Table 1. In addition, an association was found between age and sex with severe inflammatory response (*p* < 0.05).

**Table 1:** Socio-demographic characteristics (*n* = 277)

		Overall		With severe inflammatory response (*n* = 135)		Without severe inflammatory response (*n* = 142)		*p*
		n	%	n	%	n	%	
Age (in years)	20 a 39	53	19.1%	15	11.1%	38	26.8%	0.004
	40 a 59	119	43.0%	65	48.1%	54	38%	
	60 a 94	105	37.9%	55	40.7%	50	35.2%	
Sex	Men	170	61.4%	99	73.3%	71	50%	< 0.001
	Women	107	38.6%	36	26.7%	71	50%	
Civil status	Single	34	12.3%	15	11.1%	19	13.4%	0.505
	Married	195	70.4%	99	73.3%	96	67.6%	
	Divorced	18	6.5%	6	4.4%	12	8.5%	
	Widowed	30	10.8%	15	11.1%	15	10.6%	
Employment status	Unemployed	120	43.3%	55	40.7%	65	45.8%	0.469
	Employed	157	56.7%	80	59.3%	77	54.3%	
Do you belong to any religion?	No	21	7.6%	11	8.1%	10	7%	0.904
	Yes	256	92.4%	124	91.9%	132	93%	
Have you had a family member with COVID-19?	No	52	18.8%	27	20%	25	17.6%	0.722
	Yes	225	81.2%	108	80%	117	82.4%	
Has any member of your family passed away due to COVID-19?	No	192	69.3%	91	67.4%	101	71.1%	0.589
	Yes	85	30.7%	44	32.6%	41	28.9%	
Previous psychiatric diagnosis	No	258	93.1%	128	94.8%	130	91.5%	0.403
	Yes	19	6.9%	7	5.2%	12	8.5%	
Previous psychological treatment	No	252	91.0%	124	91.9%	128	90.1%	0.774
	Yes	25	9.0%	11	8.1%	14	9.9%	
Depressive symptoms	No	246	88.8%	116	85.9%	130	91.5%	0.196
	Yes	31	11.2%	19	14.1%	2	8.5%	
Anxiety symptoms	No	255	92.1%	121	89.6%	134	94.4%	0.217
	Yes	22	7.9%	14	10.4%	8	5.6%	
Somatic symptoms	No	271	97.8%	132	97.8%	139	97.9%	0.919
	Yes	6	2.2%	3	2.2%	3	2.1%	
Symptoms of post-traumatic stress	No	260	93.9%	126	93.3%	134	94.4%	0.914
	Yes	17	6.1%	9	6.7%	8	5.6%	

The *p*-value is obtained from the chi-square analysis

**Fig. 2:**
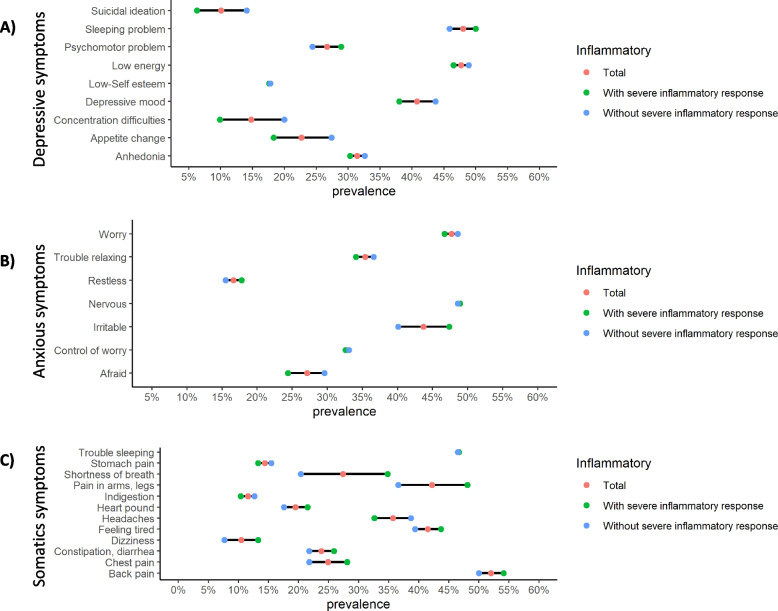
Prevalence of clinical indicators of depression, anxiety and psychosomatic symptoms

Regarding the prevalence of the overall sample, we observed a prevalence in sleep problems (48%) and low energy (47.7%) as depression indicators in the overall sample. Nervousness (48.77%), worry (47.7%) and irritability (43.7%) were the most prevalent indicators of anxiety. Back pain (52%) and trouble sleeping (46.6%) were the most common somatic symptoms. Similarities were observed among inflammatory responses. Sleeping problems (> 45.9%) and low energy (> 46.5%) were the most common depressive indicators in both groups with and without an inflammatory response. Likewise, while worry (> 46.7%) and nervousness (> 48.6%) were prevalent in both groups, irritability (47.4%) was higher in patients with severe inflammatory responses than those without inflammatory responses. Differences in somatic symptoms were observed between groups. Back pain (> 50%) and trouble sleeping (> 46.5%) were the most prevalent indicators in both samples. Pain in arms and legs (48.1%), feeling tired (43.7%), and shortness of breath indicators (34.8%) were higher in the group with inflammatory responses in comparison with the other group. The clinical indicators for each of the mental health problems by the group are summarized in Fig. 2 and detailed in Supplementary material 2.

### Relationship between variables

The findings in Table 2 indicate that correlations between scores for depression, anxiety and somatization symptoms were high for overall participants (*r* > 0.70, *p* < 0.05). A moderate relationship was also observed between symptoms of post-traumatic stress with depression, anxiety and somatic symptoms (*r* > 0.50, *p* < 0.05). A small relationship was found between the perception of COVID-19 symptoms with the other variables, in the group of all participants.

**Table 2:** Correlations between depressive symptoms, anxiety symptoms, somatic symptoms, symptoms of post-traumatic stress, and perception of physical symptom (*n* = 277)

Group	Variable	1	2	3	4	4.1	4.2	4.3	5
Overall	1. Depressive symptoms	1							
(*n* = 277)	2. Anxiety symptoms	0.77*	1						
	3. Somatic symptoms	0.73*	0.71*	1					
	4. Symptoms of post-traumatic stress disorder	0.64*	0.65*	0.67*	1				
	4.1 Intrusion	0.63*	0.63*	0.65*	0.93*	1			
	4.2 Avoidance	0.52*	0.57*	0.59*	0.92*	0.82*	1		
	4.3 Hyperarousal	0.68*	0.65*	0.69*	0.90*	0.81*	0.74*	1	
	5. Perception of physical symptoms	0.17*	0.17*	0.22*	0.11	0.13*	0.10	0.13*	1
With severe inflammatory response (NLR ≥ 6.5) (*n* = 135)	1. Depressive symptoms	1							
	2. Anxiety symptoms	0.79*	1						
	3. Somatic symptoms	0.72*	0.70*	1					
	4. Symptoms of post-traumatic stress disorder	0.62*	0.64*	0.65*	1				
	4.1 Intrusion	0.62*	0.67*	0.64*	0.92*	1			
	4.2 Avoidance	0.45*	0.54*	0.56*	0.91*	0.81*	1		
	4.3 Hyperarousal	0.68*	0.63*	0.69*	0.91*	0.83*	0.73*	1	
	5. Perception of physical symptoms	0.18*	0.08*	0.15*	0.02*	0.03	-0.01	0.05	1
Without severe inflammatory response (NLR < 6.5) (*n* = 142)	1. Depressive symptoms	1							
	2. Anxiety symptoms	0.76*	1						
	3. Somatic symptoms	0.73*	0.72*	1					
	4. Symptoms of post-traumatic stress disorder	0.66*	0.65*	0.69*	1				
	4.1 Intrusion	0.64*	0.60*	0.66*	0.93*	1			
	4.2 Avoidance	0.59*	0.60*	0.61*	0.92*	0.82*	1		
	4.3 Hyperarousal	0.67*	0.66*	0.68*	0.88*	0.79*	0.75*	1	
	5. Perception of physical symptoms	0.17*	0.27*	0.29*	0.21*	0.23*	0.23*	0.22*	1

The relationship was assessed using Spearman’s coefficient

^*^p < 0.05: significant correlation between variables

A differential analysis on the strength of the correlation between participants with and without severe inflammatory response was conducted. Among participants without inflammatory response the relationship between post-traumatic stress and symptom perception was small and significant (*r* > 0.20, *p* < 0.05). However, in the group of inflammatory responders, this same correlation was not significant. The strength of the relationship between the other variables did not change.

### Structural regression model

The general model that includes individuals with and without severe inflammatory response identified an optimal overall fit, reaching adequate goodness-of-fit indices in all the indexes evaluated (see Table 3). The proposed model explains 85% of the variance of depressive symptoms.

**Table 3:** Goodness-of-fit indices of the structural regression model

Model	n	Χ^2^	Χ^2^ /df	CFI	TLI	RMSEA [90% CI]	SRMR	R^2^
Overall	277	1963.3	1.61	0.984	0.984	0.047 [0.043—0.051]	0.085	0.850
With severe inflammatory response	135	1667.8	1.37	0.971	0.971	0.053 [0.046—0.059]	0.109	0.837
Without severe inflammatory response	142	1552.1	1.28	0.960	0.959	0.044 [0.037—0.051]	0.128	0.898

*Χ*^*2*^ chi-square, *CFI* comparative fit index, *TLI* Tucker-Lewis's index, *RMSEA* root mean square error of approximation, *CI* confidence intervals, *SRMR* standardized root mean square. *R*^*2*^ coefficient of determination, Degrees of freedom = 1,216

**Fig. 3:**
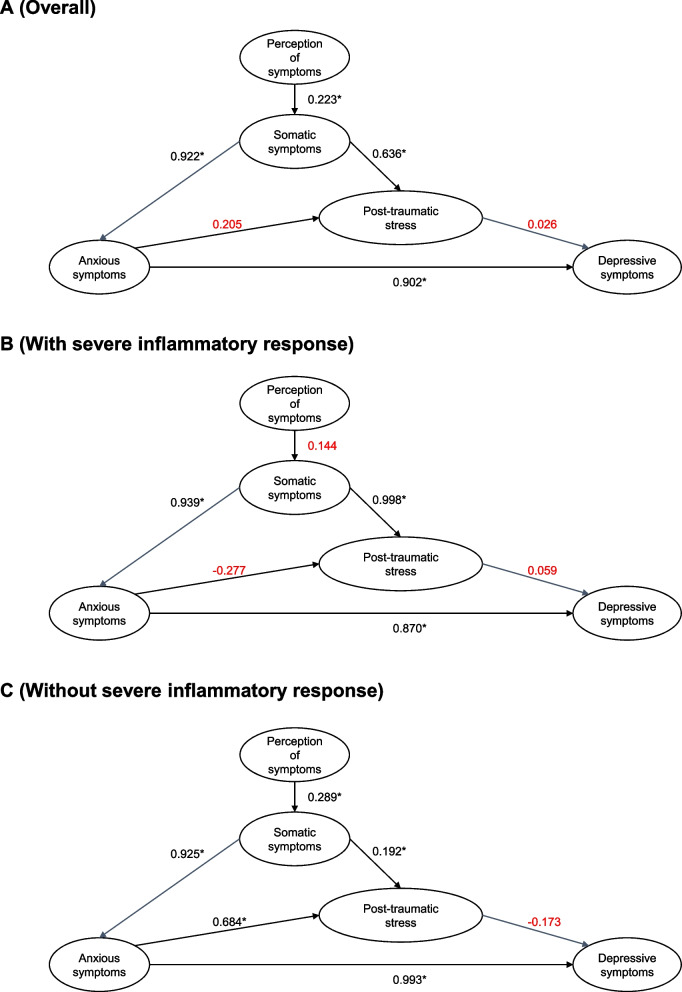
Path Analysis. Note: **A** Overall participant. **B** Participants with severe inflammatory response. **C** Participants without severe inflammatory response. The model was estimated with the WLSMV method. Values in red are not significant. *p < 0.05

In the general model (see Fig. 3A), the perception of symptoms influenced somatic symptoms (β = 0.223, *p* < 0.05). In addition, somatic symptoms influenced anxiety symptoms (β = 0.922, *p* < 0.05) and post-traumatic stress symptoms (β = 0.623, *p* < 0.05). A non-significant relationship was found between anxiety and post-traumatic stress symptoms (β = 0.205, *p* = 0.262) and stress-post traumatic stress with depressive symptoms (β = 0.026, *p* = 0.727). Finally, the relationship between anxiety and depressive symptoms was high and significant (β = 0.902, *p* < 0.05).

When analyzing separately the overall performance of the models for participants with and without inflammatory response, both models presented adequate goodness-of-fit indices and explained more than 83% of the variance of depressive symptoms. However, the SRMR values are high, possibly due to the small sample size (*n* < 200) (see Table 3).

The specific assessment of the relationships of the proposed models identified different performances for participants with a severe inflammatory response (see Fig. 3B) and for participants without a severe inflammatory response (see Fig. 3C). The relationship between perception of COVID-19 symptoms and somatic symptoms was found to be significant for this group of participants without severe inflammatory response (β = 0.289, *p* < 0.05), but the relationship was not significant in the group of participants with a severe inflammatory response (β = 0.289, *p* = 0.117). The relationship between symptoms of post-traumatic stress and anxiety symptoms in the group without severe inflammatory response was direct, significant, and high (β = 0.684, *p* < 0.05). However, the relationship was inverted and not significant among participants with a severe inflammatory response (β = -0.277, *p* = 0.531). A non-significant relationship was found between symptoms of post-traumatic stress and depressive symptoms in both groups (i.e., with and without a severe inflammatory response).

## Discussion

### Main findings and significance of the results

There is extensive discussion on the role of biological and psychological variables in the occurrence of depressive symptoms in patients with COVID-19. Previous studies found higher values ​​in inflammatory markers (i.e., NLR) in patients with depression, compared with the non-depression groups [25, 61]. Our research presents evidence that a model depicting the subjective perception of symptoms (psychosomatic and COVID-19 symptoms) withs anxiety and post-traumatic stress symptoms explains the presence of depressive symptoms in patients with and without severe inflammatory response in NLR who were hospitalized for COVID-19.

We used the anxiety-PTSD-depression triad as a base model and added the variables related to the subjective perception of symptoms. Our model explained 85% of depressive symptoms in the overall sample, indicating that several mood disorders occur simultaneously prior to the development of depression [67].

The model also showed that psychosomatic symptoms and anxiety have the most influence in the occurrence of depressive symptoms. The impact of these variables may be explained by biological perspectives. For instance, an increase of anxiety is associated with presence of somatic symptoms, such as headaches and shoulder and limb pain [67]. Besides, the relationship between psychosomatic symptoms and symptoms of post-traumatic stress represents a relevant effect, which can be explained due to somatic symptoms being more prevalent during periods of stress [68].

### Contrasting findings with existing literature

#### Prevalence and indicators

In our study, the most prevalent mental health problems were anxiety (11.2%), depression (7.1%) and PTSD (6.1%). Similarly, a previous study conducted in Peru reported the prevalence for depression (12.1%), anxiety (8.4%), and PTSD (10.5%) in health care-workers during the pandemic [24]. The prevalence reported in systematics reviews indicated higher values than our study showed for depression (45%), anxiety (47%), and PTSD (16 to 22.6%) [4, 69, 70]. These differences among researchers could be explained due to the different socio-demographic compositions, different study designs, and measurement instruments used, which may influence the degree of prevalence. Even so, results evidenced that the patients with COVID-19 present several mental health problems at the same time. This could mean that, during the treatment, patients may develop multiple related psychiatric diseases which form a mutually influential symptom network, in turn, influencing their recovery [71].

Sleep problems were one of the most frequent indicators of mental health problems. This relationship was previously reported in a systematic review, in which sleep problems were associated with higher levels of mental health problems (i.e., anxiety and depression) among mental health care-workers, general population, and COVID-19 patients [72]. Also, this indicator has been found to be prevalent in healthcare workers even before COVID-19 [72]. A possible explanation for the prevalence of sleep problems could be fear of COVID-19. Due to worries about the disease, patients may struggle with sleep and consequently develop insomnia [73]. Moreover, if the individual cannot manage the fear for a specific time, they may experience mental health problems (e.g., depression, anxiety) [74]. We also found that low energy had the second highest, which is a comorbid symptom of many psychiatric problems in patients with COVID-19 [75]. In addition, low energy could be caused by lack of sleep or one of the other mental health problems such as depression.

The main indicators related to anxiety were nervousness, irritability, and worry. This finding was also reported in other studies in individuals with COVID-19, with the most common symptoms of anxiety being insomnia, irritability, restlessness, and excessive worrying [76]. As previously mentioned, being hospitalized, patients may experience fear of COVID-19 and worries related to their health, family, financial issues, and environmental conditions (e.g., isolation, uncertainty about the evolution of the disease). In our study, somatic complaints (i.e., backache, arms, and legs pain, and feeling exhausted) were most prevalent. This may be due to similarities between somatic complaints and physiological symptoms of COVID-19.

### Structural equation model and relationship between variables

Previous studies of predicted models identified an impact negative of COVID-19 on mental health problems (i.e., depression, anxiety, stress, fear of COVID-19, among others). Two studies proposed models in which fear of COVID is significantly and positively related to depression, anxiety, and insomnia [77, 78]. Moreover, epidemiological studies reported the SARS-CoV-2 virus could lead to systems immune changes, which in turn could reflect in mental health problems. These psychiatric outcomes can be influenced by other factors as well (i.e., biological, factor social isolation, adverse effects of treatments, etc.) [12, 79]. However, a small number of studies has proposed predictive models about the relationship of biological responses with these mental clinical problems. For example, a study, using SEM, proposed a model that cortisol (as an indicator of Hypothalamic pituitary adrenal) predicts depression, which predicts circulating pro-inflammatory cytokines (IL-2, IL-6, TNF-α) In patients diagnosed with chronic fatigue syndrome (CFS) [80]. Another study explored the relationship between biological factors (i.e., sex, disease duration, self-perceived illness severity, and inflammatory markers) and mental health status in inpatients with COVID-19. In the SEM, inflammatory markers (i.e., NLR, IL-1β as observed variables) and mental health (i.e., insomnia, depression, and anxiety as observed variables) were set as latent variables. Results indicated the inflammatory markers had a significant and direct effect on mental health. Moreover, the disease duration and inflammatory markers indirectly influenced mental health, through self-perceived illness severity as a mediator [25]. These findings suggest that inflammatory responses could be related to psychological disorders.

Following this hypothesis, studies have found a heterogeneous influence of NLR on psychological mental problems. First, one study, using regression analysis, demonstrated the influence of NLR markers on both the prevalence of depression and anxiety in Chinese patients with gastric cancer [81]. In contradiction to this finding, a multi-linear regression study showed a weak association between inflammatory biomarkers and depression in a three-month cohort of stroke patients [82]. As similar immune responses exist in both COVID-19 infection and mood disorders, they may share biological response as well. Both states induce the production of abnormal levels of cytokines, chemokines, and other inflammatory mediators [83], showing a hyperinflammatory state [84]. While patients with mental health problems showed high levels of biomarkers [17], a meta-analysis, with 16 studies, evidenced higher counts of biomarkers (i.e. IL-6, CRP, PCT, among others) in severe cases of COVID-19 [85].

Another interesting result was the high influence of anxiety on depression in all three models. This finding is in accordance with other studies which evidenced that anxiety symptoms had a direct and significant relationship with depression. One study that proposed a model of the triad fear-anxiety-stress in the development of depression symptoms in pandemic disease symptoms in health workers, indicated that the fear of COVID-19, anxiety and post-traumatic symptoms explains depression symptoms. The SEM demonstrated that anxiety was the most influential variable in depression symptoms in comparison with post-traumatic stress [24]. Preceding COVID-19, researchers have shown that anxiety may contribute directly or as mediating variables in depression. These results show how the different variables (i.e., stress, self-esteem, stressful negative events) influences depression, where increases in anxiety may lead to increases in depression [86, 87]. In sum, these findings suggest that the role of anxiety in the occurrence of depressive symptoms is significant and is even maintained in the COVID-19 pandemic. Anxiety is a common adaptive response against threatening situations, which could be increased due to factors such as stress or fear and could trigger prolonged anxiety. Thus, pathological anxiety can affect functioning in the daily routine of patients, which in turn may cause or be comorbid with other mental disorders such as depression [88].

Another result was the influence of the PTSD variable on depression. Our results demonstrated that PTSD symptoms do not present a significant influence on depression in hospitalized patients with and without severe inflammatory markers. This finding might be related to the PTSD symptoms changes over time. Other studies have found the different prevalence of PTSD symptoms in each stage of COVID-19 disease (i.e., recovering from COVID-19 infection, being quarantined) [89, 90]. Likewise, another reason could be the similarity between our variables. There are studies that report a high association between PTSD and somatic symptoms. Findings support the fact that somatic symptoms may be related to the patient’s psychophysiological dysregulation and lead to psychological symptoms (e.g., PTSD) [91, 92].

### Implications in public health and making decisions

Our findings provide a theoretical model, which may help establish policies to prevent depression among inpatients with COVID-19. Specifically, the model revealed that somatic and anxiety symptoms are the most relevant predictors to develop depression. Health workers could employ screening measures for anxiety and somatic symptoms to prioritize the care of patients with high levels in these conditions, and thus avoid possible cases of depressive symptoms. It is a necessity because Peru is one of the countries that reported worse mental health levels in the world during the pandemic [93] Also, the prevalence of depression in 2020 was five times higher than previous years [94].

Interventions to reduce symptoms of anxiety, fear and worry in hospitalized patients could prevent subsequent cases of mental illness [95]. For example, telephone-based interventions also have been useful to reduce symptoms of anxiety and depression, providing psychological support, information about the process of the disease and promoting a sense of emotional stability [96, 97]. Thus, the implementation of telephones during hospitalizations could be a strategy to prevent psychological problems in hospital isolated patient so and could be a facility for patients to have access to make calls or send messages to their relatives.

### Strengths and limitations

This study has limitations that should be mentioned. First, some patients did not have inflammatory markers recorded, so they were excluded. This exclusion could lead to an information bias. Second, NLR was evaluated as the only inflammatory measure. However, using inflammatory markers to assess inflammatory response is not considered a gold standard. Therefore, doing so may have caused errors when grouping participants into those with and without a severe inflammatory response. Third, this study has a cross-sectional design, thus we cannot infer causality in the interpretation of the findings. Fourth, we employed self-reported measures, which may have been influenced by social desirability or memory bias. Fifth, the data includes a single hospital in a Peruvian city. Therefore, results should not be generalized to other cities or contexts. Sixth, we used a validated scale such as the IES-R to measure PTSD, however, IES-R does not include the entire concept of PTSD. The Diagnostic and Statistical Manual of Mental Disorders (DSM–5) considers four dimensions and the IES-R only assesses three of these dimensions. This could imply a partial evaluation of the symptoms of PTSD. Finally, other confounding variables were not considered, such as fear of COVID-19 [24] and coping [98]. Thus, it is possible that the model is partial or influenced by other variables.

On the other hand, our study has three main strengths. This investigation presents a larger sample compared to previous studies evaluating hospitalized patients [25, 99]. We also employed structural equation modelling, which allowed us to assess several variables simultaneously. Moreover, to our knowledge, this is the first study that provides a framework of biological and psychological variables that explain depressive symptoms as an outcome in the context of the COVID-19.

### Conclusions and recommendations

Results demonstrated that our model of mental health variables may explain depression in hospitalized patients of COVID-19 from a third-level hospital in Peru. In the model, perception of symptoms influences somatic symptoms, which influence both anxiety symptoms and symptoms of post-traumatic stress. Thus, anxiety symptoms could directly influence depressive symptoms or through PTSD symptoms. Additionally, our model was found to have a good overall fit and explained more than 83% of the depressive symptoms.

Regarding clinical indicators, patients presented a high prevalence of depression, anxiety, and psychosomatic indicators. Our findings could be useful to decision-makers for the prevention of depression, such as to encourage the use of screening tools (i.e., perception of symptoms, somatic symptoms, anxiety) that may sooner identify patients vulnerable to depression.

## Supplementary Information

**Additional file 1. Supplementary material 1. **Psychometric properties of the scales used (n=277).

**Additional file 2. Supplementary material 2. **Values of the prevalence of clinical indicators of depression, anxiety and psychosomatic symptoms (n=277).

## Data Availability

The datasets generated and analysed during the current study are not publicly available due their containing information that could compromise the privacy of research participants but are available from the corresponding author on reasonable request.
