# Supplementary material for: An explanatory model of depressive symptoms from anxiety, post-traumatic stress, somatic symptoms, and symptom perception: the potential role of inflammatory markers in hospitalized COVID-19 patients
Source: BMC Psychiatry. 2022 Oct 10;22:638. doi: 10.1186/s12888-022-04277-4 (PMC9548421; doi:10.1186/s12888-022-04277-4)
Supplement: Supplementary file 1 — Additional file 1. Supplementary material 1. Psychometric properties of the scales used (n=277). [file 12888_2022_4277_MOESM1_ESM.pdf]

Supplementary material 1. Psychometric properties of the scales used (n=277).

Scale	Variable	X^2 (gl)	CFI	TLI	SRMR	RMSEA [90%CI]	α	ω
PHQ-9	Depressive symptoms	59.1 (27)	0.988	0.984	0.044	0.066 [0.043-0.088]	0.88	0.90
GAD-7	Anxiety symptoms	12.4 (14)	0.998	0.998	0.025	0.001 [0.000-0.052]	0.87	0.88
PHQ-12	Somatic symptoms	103.8 (54)	0.972	0.966	0.078	0.058 [0.041-0.074]	0.83	0.86
IES-R	Symptoms of post-traumatic stress	588.6 (206)	0.968	0.964	0.082	0.082 [0.074-0.090]	Global = 0.95;<br>Intrusion = 0.87;<br>Avoidance = 0.90;<br>Hyperarousal = 0.83	Global = 0.97;<br>Intrusion = 0.90;<br>Avoidance = 0.91;<br>Hyperarousal = 0.86

Note: PHQ-9 = Patient Health Questionnaire-9. GAD-7 = General Anxiety Disorder-7. PHQ-15 = Patient Health Questionnaire-15. IES-R = Impact of Events Scale-Revised. $X^{2=}$ chi-square. CFI = comparative fit index. TLI = Tucker-Lewis's index. RMSEA = root mean square error of approximation. CI = confidence intervals. SRMR = standardized root mean square. gl = Degrees of freedom.
