# Supplementary material for: An explanatory model of depressive symptoms from anxiety, post-traumatic stress, somatic symptoms, and symptom perception: the potential role of inflammatory markers in hospitalized COVID-19 patients
Source: BMC Psychiatry. 2022 Oct 10;22:638. doi: 10.1186/s12888-022-04277-4 (PMC9548421; doi:10.1186/s12888-022-04277-4)
Supplement: Supplementary file 2 — Additional file 2. Supplementary material 2. Values of the prevalence of clinical indicators of depression, anxiety and psychosomatic symptoms (n=277). [file 12888_2022_4277_MOESM2_ESM.pdf]

Supplementary material 2. Values of the prevalence of clinical indicators of depression, anxiety and psychosomatic symptoms (n=277).

Variable	Item	Indicator	Overall	With severe inflammatory response (NLR≥ 6.5)	Without severe inflammatory response (NLR< 6.5)
	PHQ-9_1	Anhedonia	31.4	30.3	32.6
	PHQ-9_2	Depressive mood	40.8	38.0	43.7
	PHQ-9_3	Sleeping problem	48.0	50.0	45.9
	PHQ-9_4	Low energy	47.7	46.5	48.9
Depression	PHQ-9_5	Appetite change	22.7	18.3	27.4
	PHQ-9_6	Concentration difficulties	14.8	9.9	20.0
	PHQ-9_7	Psychomotor problem	26.7	28.9	24.4
	PHQ-9_8	Low-Self esteem	17.7	17.6	17.8
	PHQ-9_9	Suicidal ideation	10.1	6.3	14.1
	GAD-7_1	Nervous	48.7	48.9	48.6
	GAD-7_2	Control of worry	32.9	32.6	33.1
	GAD-7_3	Worry	47.7	46.7	48.6
Anxiety	GAD-7_4	Trouble relaxing	35.4	34.1	36.6
	GAD-7_5	Restless	16.6	17.8	15.5
	GAD-7_6	Irritable	43.7	47.4	40.1
	GAD-7_7	Afraid	27.1	24.4	29.6
	PHQ-15_1	Stomach pain	14.4	13.3	15.5
	PHQ-15_2	Back pain	52	54.1	50
	PHQ-15_3	Pain in arms, legs	42.2	48.1	36.6
	PHQ-15_5	Headaches	35.7	32.6	38.7
	PHQ-15_6	Chest pain	24.9	28.1	21.8
Somatic symptoms	PHQ-15_7	Dizziness	10.5	13.3	7.7
	PHQ-15_9	Heart pound	19.5	21.5	17.6
	PHQ-15_10	Shortness of breath	27.4	34.8	20.4
	PHQ-15_12	Constipation, diarrhea	23.8	25.9	21.8
	PHQ-15_13	Indigestion	11.6	10.4	12.7
	PHQ-15_14	Feeling tired	41.5	43.7	39.4
	PHQ-15_15	Trouble sleeping	46.6	46.7	46.5

Note: PHQ-9 = Patient Health Questionnaire-9. GAD-7 = The General Anxiety Disorder- 7. PHQ-15 = The Patient Health Questionnaire-15.
